# Oral health attitudes and behavior among undergraduate students

**DOI:** 10.1192/j.eurpsy.2022.2287

**Published:** 2022-09-01

**Authors:** E. Nikolaev, S. Petunova, N. Dakdaki

**Affiliations:** 1 Ulianov Chuvash State University, Social And Clinical Psychology Department, Cheboksary, Russian Federation; 2 Ulianov Chuvash State University, Medical Faculty, Cheboksary, Russian Federation

**Keywords:** Oral behavior, Undergraduate students, health attitudes, oral health

## Abstract

**Introduction:**

Oral health behavior is based on an acquired experience and cultural traditions. University education may smooth out cultural differences in oral health practice.

**Objectives:**

Our goal is to study self-reported oral health attitudes and behavior of university students and the cultural basis for it.

**Methods:**

We used the English version of the Hiroshima University Dental Behavioral Inventory to carry out an online survey of 136 university students of Morocco and Russia.

**Results:**

Over half of the students (60.3%) do not feel anxious when visiting a dentist. Most of them take care of their gums (41.2%), teeth color (49.3%) and the degree of their cleanness (38.2%). The overwhelming majority of the students brush their each tooth very thoroughly (62.5%), they regularly examine their teeth in the mirror after brushing them (90.4%). They are well aware that tooth brushing alone cannot prevent a gum disease (63.2%), and they feel concerned about the possibility of having bad breath (73.6%). At the same time, over half of the students (61.7%) put off their visit to a dentist until they have a toothache, which is a negative behavioral factor. We did not reveal any gender or cultural differences between the students of the two countries, which can be regarded as a universalization factor of oral health behavior in young people who get higher education in universities.

**Conclusions:**

The majority of the surveyed Russian and Moroccan university students have similar patterns of oral health attitudes and behavior. This assumption needs verification on a larger sample of students.

**Disclosure:**

No significant relationships.

